# From common origin of life to seeking common path of diseases – a new paradigm of translation

**DOI:** 10.1186/1479-5876-10-S2-A26

**Published:** 2012-10-17

**Authors:** Fei Xiao, James Williams

**Affiliations:** 1 Cinkate Pharmaceutical Research Institute, Shanghai, China; 2 Department of Surgery, University of Chicago, Chicago, Illinois, USA

## 

More than 13 billion years ago, the universe emerged and continued expending after the Big Bang. 4.6 billion Years ago, our earth was formed in solar system with no oxygen and no any signs of life. 3.8 billion years ago, primitive elements of life of RNA and DNA were accidently formed within rocky vent in deep water of Atlantic Ocean. From single cell to multiple cell life, and to multi-organ species, it took billions years. The ocean turned blue, the land got green and oxygen fills our air. The life started move on to the land. The lives were evolving for adapting with the surrounding environment and the forms of life were diversified.

However, inside each basic block of life, the cell, our common ancestor inscribed the deep common marks. From genome to protein, to signal transduction, to organelles, to cell membrane, we all life on earth are in a big family. This is the basis for our human who benefit from using our sister species to do medical experiments.

The fates of life are also in common. Through the journey of birth, growth, aging and death, we all reach the same destiny. Cells are subject to the fates of senescence, mutation, apoptosis, and necrosis. The clock of life is tickling down in according with the shortening of telomeres.

Once our tissue is injured, regardless of either from inside of autoimmune response or metabolic disorder, or from outside micro-organism invasion or traumatic damage, our repairing potential is intrigued, which induces the common reactions include inflammatory cells infiltration, extracellualr matrix formation. Once the damage is repaired, the inflammatory cells commit programed death, the apoptosis, and healing is achieved. If the injure continues and inflammation persists, the fibrosis and scaring process dominate, and then, the organ lost function and slides to end stage.

Understanding the nature of common origin of life and exploring common path of disease would help us to see a big picture about life science, and lead to generate big ideas to deal with disease. With integration of multi-discipline collaborations, the common targets for treatment of diseases are being identified and right strategies can be developed. Efficiently developing new drugs and wisely applying old ones, rationally combining biologic with small molecule, focusing on both physical and mental would speed successes in translation and achieve the healthy lives in human being, and even dial the life clock back.

